# Guideline-concordant administration of prothrombin complex concentrate and vitamin K is associated with decreased mortality in patients with severe bleeding under vitamin K antagonist treatment (EPAHK study)

**DOI:** 10.1186/cc13843

**Published:** 2014-04-24

**Authors:** Karim Tazarourte, Bruno Riou, Benjamin Tremey, Charles-Marc Samama, Éric Vicaut, Bernard Vigué

**Affiliations:** 1 Service d’Aide Médicale Urgente 77 and Department of Emergency, Centre Hospitalier Melun, Melun, France; 2 Department of Emergency Medicine and Surgery, Centre Hospitalier Universitaire Pitié-Salpêtrière, Assistance Publique-Hôpitaux de Paris, INSERM UMR 1166, Université Pierre et Marie Curie-Paris VI, Paris, France; 3 Department of Anesthesiology, Centre Médico-Chirurgical Ambroise Paré, Neuilly-sur-Seine, France; 4 Department of Anesthesia and Intensive Care Medicine, Hôtel Dieu and Cochin University Hospitals, Assistance Publique-Hôpitaux de Paris, Université Paris Descartes, Paris, France; 5 Department of Biostatistics, Centre Hospitalier Universitaire Lariboisière, Assistance Publique-Hôpitaux de Paris, Université Denis Diderot, Paris, France; 6 Department of Anesthesiology and Critical Care, Centre Hospitalier Universitaire de Bicêtre, Assistance Publique-Hôpitaux de Paris, Le Kremlin-Bicêtre, France

## Abstract

**Introduction:**

In vitamin K antagonist (VKA)-treated patients with severe hemorrhage, guidelines recommend prompt VKA reversal with prothrombin complex concentrate (PCC) and vitamin K. The aim of this observational cohort study was to evaluate the impact of guideline concordant administration of PCC and vitamin K on seven-day mortality.

**Methods:**

Data from consecutive patients treated with PCC were prospectively collected in 44 emergency departments. Type of hemorrhage, coagulation parameters, type of treatment and seven-day mortality mortality were recorded. Guideline-concordant administration of PCC and vitamin K (GC-PCC-K) were defined by at least 20 IU/kg factor IX equivalent PCC and at least 5 mg of vitamin K performed within a predefined time frame of eight hours after admission. Multivariate analysis was used to assess the effect of appropriate reversal on seven-day mortality in all patients and in those with intracranial hemorrhage (ICH).

**Results:**

Data from 822 VKA-treated patients with severe hemorrhage were collected over 14 months. Bleeding was gastrointestinal (32%), intracranial (32%), muscular (13%), and “other” (23%). In the whole cohort, seven-day mortality was 13% and 33% in patients with ICH. GC-PCC-K was performed in 38% of all patients and 44% of ICH patients. Multivariate analysis showed a two-fold decrease in seven-day mortality in patients with GC-PCC-K (odds ratio (OR) = 2.15 (1.20 to 3.88); *P* = 0.011); this mortality reduction was also observed when only ICH was considered (OR = 3.23 (1.53 to 6.79); *P* = 0.002).

**Conclusions:**

Guideline-concordant VKA reversal with PCC and vitamin K within eight hours after admission was associated with a significant decrease in seven-day mortality.

## Introduction

In developed countries, up to 2% of the general population receives chronic oral anticoagulation with vitamin K antagonists (VKA) [1]. The rate of severe bleeding complications is above 3.8% per person-year and the associated mortality rate ranges between 10 to 60%, especially in case of intracranial hemorrhage (ICH), in the first week following the onset of the hemorrhage [1-4]. In case of severe hemorrhage, the two main predictors of poor prognosis are over anticoagulation, that is, an international normalized ratio (INR) above the therapeutic range, and late VKA reversal (over eight hours after presentation) [5,6].

For several decades the evidence supporting appropriate clinical use of VKA antidotes has remained scarce [7]. In a recent randomized clinical trial, Sarode *et al*. demonstrated the superiority of prothrombin complex concentrate (PCC) versus fresh frozen plasma (FFP) to normalize coagulation within one hour (62.2 versus 9.6%; *P* <0.05) [8]. The role of vitamin K in association with PCC, in maintaining the normalized coagulation over six hours has been emphasized [9]. The importance of rapid hemorrhage control has led international guidelines to recommend the use of PCC rather than FFP [10-12]. For complete VKA reversal, all guidelines recommend an infusion of PCC (at least 20 IU/kg factor IX equivalent) related to admission INR value in combination with at least 5 mg of vitamin K to rapidly achieve a post-reversal INR ≤1.5 and maintain a normal coagulation profile over six hours. So as to save time, French guidelines suggest, on the basis of a previous study, the possibility of administering a probabilistic single-regimen dose of 25 IU/kg of PCC and 10 mg of vitamin K as soon as a severe hemorrhage is diagnosed [9,10]. In all situations, post-reversal INR must be measured 30 minutes after the infusion to evaluate the efficacy of the treatment and make any necessary adjustments thereafter, and measured six hours later to control the efficacy of vitamin K [10-12]. French guidelines were published in 2008 and have become a standard of care for the management of these patients in France [10]. Treatment time frames were not specified in the international guidelines and the effect of early reversal on mortality has not been studied. At a time when new oral anticoagulant agents without specific antidotes are emerging as promising alternatives to VKAs, it appears important to better define the prognosis benefit of anticoagulant reversal in the management of patients on oral anticoagulants with severe hemorrhage [13].

The aim of this observational study was to evaluate the impact of guideline-concordant administration of PCC and vitamin K (GC-PCC-K) on early mortality in VKA-treated patients with severe hemorrhage. We tested the hypothesis that GCA-PCC-K is associated with improved early outcome.

## Methods

### Patients and procedures

This prospective observational study was conducted from May 2009 to June 2010 in 44 emergency department hospitals in France. The protocol was approved by the Institutional Review Board (Comité d’évaluation de l’éthique des projets de recherche biomédicale, CEERB, Paris, France) on 5 June 2009. Electronic database permissions were obtained from the Comité consultatif sur le traitement de l’information en matière de recherche dans le domaine de la santé (CCTIRS) on 15 January 2009 and from the Commission nationale de l’informatique et des libertés (CNIL) on 3 April 2009. Because there was no randomization and only standard of care was performed, the Institutional Review Board waived the need for informed patient consent. Academic (*N* = 29) and general (*N* = 15) hospitals located in rural or urban areas were included. Strengthening the Reporting of Observational Studies in Epidemiology (STROBE) guidelines for reporting observational studies were followed [14].

In each center consecutive VKA-treated patients, aged over 18 years with the exception of pregnant women, with a severe hemorrhage were included in the study. Hemorrhage was defined as severe if at least one of the following criteria was present: 1) externalized bleeding that cannot be stopped by applying conventional measures; 2) hemodynamic instability defined by systolic arterial blood pressure (SAP) <90 mmHg, or signs of shock; 3) need for an emergency procedure to stop bleeding; 4) need for red blood cell transfusion; 5) life-threatening bleeding or bleeding that compromises function, for example, intracranial or intraspinal hemorrhage, intraocular or retro-orbital bleeding, hemothorax, hemoretroperitoneum, hemopericardium, deep-muscle hematoma and/or neural compression syndrome, acute gastrointestinal bleeding and hemarthrosis [10].

Hemorrhages were classified into four categories: 1) gastrointestinal hemorrhages; 2) ICH; 3) deep-muscle and subperitoneal hematomas; and 4) “others”, including hemothorax, hemopericardium as well as hemorrhages for which anticoagulant reversal is not systematically required but may be urgently needed in case of shock or transfusion (for example, epistaxis or hematuria).

The data collected were patient characteristics, medical treatments, indication, duration and type of VKA treatment, history of thrombotic and bleeding events, Glasgow coma scale score (GCS), SAP, admission and post-reversal INR, and reversal treatment data (time from admission to reversal, PCC dose, vitamin K dose, surgical or radiological intervention, number of transfused red blood cell units, FFP units and platelet concentrates). GCS was divided into three strata: GCS ≤8 (severe), 8 < GCS ≤13 (moderate), and GCS >13 (mild). SAP was also divided into three strata: <90 mmHg (hypotension), 90 ≤ SAP ≤140 mmHg, and >140 mmHg (hypertension). Patients with an INR under 1.5 at admission were considered as having a normal coagulation profile. An INR between 1.5 and 4 was considered within the therapeutic range (including in patients with mechanical heart valves) and an INR >4 above the therapeutic range. When post-reversal INR was obtained, the value and the time from admission to the end of PCC administration were recorded. To provide biological evidence of coagulation normalization following reversal, the proportion of patients with a post-reversal INR ≤1.5 was determined. A four-factor PCC widely available in French hospitals was used as Kaskadil^©^ until September 2009 and Kanokad^©^ (Laboratoire Français du Biomédicament LFB, Les Ulis, France) or Octaplex^©^ (Octapharma, Swizerland). These products were a human plasma-derived four-factor PCC, including factors II, VII, IX and X, and have undergone detergent treatment and nanofiltration (Kanokad^©^ and Octaplex^©^) for viral inactivation. These products also contain proteins C and S, two natural anticoagulant vitamin K dependent factors. For these two PCC, 1 ml corresponds to 25 IU of Factor IX. VKA reversal was considered guideline-concordant if a PCC dose ≥20 IU/kg and a vitamin K dose ≥5 mg were administered. Although no time frame was specified in the guidelines, we considered treatment administration within eight hours after hospital admission as a criterion for good practice, based on the median time from admission to treatment observed in a previous study [5,15].

The VKA reversal strategy was classified into five levels:

Level 0: no treatment or any treatment administered over eight hours after admission at the hospital

Level 1: PCC dose <20 IU/kg, vitamin K dose ≥5 mg and time to treatment ≤8 hours

Level 2: PCC dose ≥20 IU/kg, vitamin K dose <5 mg and time to treatment ≤8 hours

Level 3: PCC dose ≥20 IU/kg, vitamin K dose ≥5 mg and time to treatment ≥4 hours and <8 hours

Level 4: PCC dose ≥20 IU/kg, vitamin K dose ≥5 mg and time to treatment <4 hours

GC-PCC-K was considered only for levels 3 and 4. Administration of FFP was not considered as an appropriate treatment according to the guidelines [10-12]. Patients were classified automatically according to this algorithm. If data were missing, the case was reviewed and classified by two experts designated by the scientific committee. In case of disagreement, a third expert was assigned to definitely categorize the patient. Agreement between experts was calculated (kappa score).

The primary outcome was seven-day mortality. Secondary outcome measures included thromboembolic events and hemorrhage recurrences within seven days.

### Statistical analysis

Data are presented as mean ± standard deviation (SD), median (25^th^ to 75^th^ percentiles) or number (percentage).

The sample size was determined based on a preliminary study that provided a first estimate of mortality in the absence of appropriate reversal [16]. In the present study the sample size was calculated at *N* = 750 patients in order to allow a 80% power to detect (with a two-sided 5% α risk) a difference in mortality corresponding to 20% in the absence versus 12% in the presence of appropriate reversal, estimating that approximately 30% of the studied patients would receive appropriate reversal.

Factors associated with seven-day mortality were identified through univariate analysis (t-tests or χ^2^ test). A multivariate analysis was then performed to identify independent factors in the overall study population and in the subgroup of patients with ICH. Variables included in the multivariate analysis were those significant (*P* <0.05) in univariate analysis and treatment appropriateness. Calibration of the multivariate model was assessed using the Hosmer-Lemeshow test and discrimination using c-statistics. To perform an internal validation, we used a bootstrapping procedure (*N* = 500 bootstrap samples) to evaluate optimism of the parameters of models and calculated the odds ratio (OR) associated with inappropriate treatment and its 95% confidence interval (CI). The difference between this OR and the observed OR in the cohort was defined as the optimism.

In view of the high mortality associated with ICH compared with other types of hemorrhages, we also prespecified a subgroup analysis of patients with ICH [5].

All *P-*values were two-tailed and statistical significance was set at *P* <0.05.

## Results

**Figure 1:**
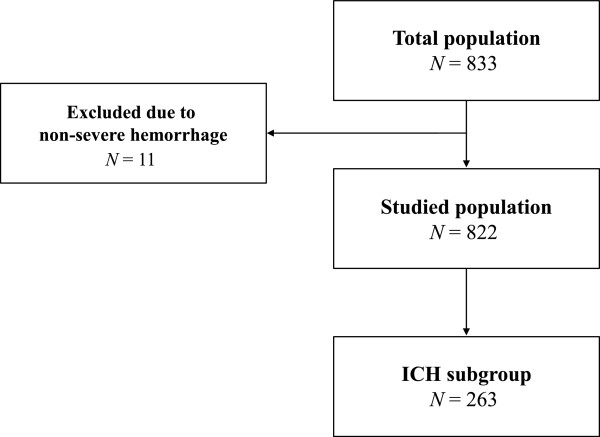
Study flow chart.

The study flow chart is shown in Figure 1. A total of 833 patients were included in the study (detailed information on patient recruitment in the participating centers is provided in the Acknowledgment section). Eleven patients were excluded because they did not meet the criteria for severe hemorrhage. At Day 7, mortality was recorded for all patients included. Therefore, the analyzed population comprised 822 patients, including 262 (32%) patients with ICH. The main characteristics of the whole cohort and patients with ICH are shown in Table 1. A total of 110 patients (13%) died within seven days, including 86 ICH patients, representing 10% of the overall population and 33% of the ICH subgroup. Deaths in the ICH subgroup accounted for 78% of deaths in the overall population (Table 1).

**Table 1:** Main characteristics of the cohort

**Variable**	**All patients (*N*** **= 822)**	**Alive (*N*** **= 712)**	**Dead (*N*** **= 110)**	***P***
Age (yrs)	77 ± 11	77 ± 11	80 ± 9	0.020
≤65	129 (16%)	119 (17%)	10 (9%)	0.033
66 to 75	144 (17%)	131 (18%)	13 (12%)	
76 to 85	368 (45%)	310 (44%)	58 (53%)	
>85	181 (22%)	152 (21%)	29 (26%)	
Men	468 (57%)	408 (57%)	60 (55%)	0.587
Women	354 (43%)	304 (43%)	50 (45%)	
Type of VKA treatment				
Fluindione	631 (77%)	535 (75%)	96 (87%)	0.005
Acenocoumarol	99 (12%)	91 (13%)	8 (7%)	0.098
Warfarin	85 (10%)	80 (11%)	5 (5%)	0.032
Missing data	7	6	1	
Indication for VKA treatment*				
Atrial fibrillation	555 (68%)	470 (66%)	85 (78%)	0.013
Venous thromboembolic disease	156 (19%)	143 (20%)	13 (12%)	0.043
Prosthetic heart valve	102 (12%)	89 (13%)	13 (12%)	0.850
Others	79 (10%)	66 (9%)	13 (12%)	0.386
Duration of VKA treatment				
<1 year	137 (17%)	124 (18%)	13 (13%)	0.223
1 to 5 years	236 (30%)	207 (30%)	29 (29%)	0.846
>5 years	426 (53%)	367 (52%)	59 (58%)	0.272
Missing data	23	14	9	
Antiplatelet treatment**	153 (19%)	132 (19%)	21 (19%)	0.895
Aspirin	125 (15%)	107 (15%)	18 (16%)	0.688
Clopidogrel	34 (4%)	31 (4%)	3 (3%)	0.694
Missing data	1	1	0	
History of severe hemorrhage**	103 (13%)	93 (13%)	10 (10%)	0.331
Missing data	10	3	7	
Type of hemorrhage				
Intracranial	262 (32%)	176 (25%)	86 (78%)	<0.001
Gastrointestinal	264 (32%)	253 (36%)	11 (10%)	<0.001
Deep-muscle hematomas	107 (13%)	103 (15%)	4 (4%)	<0.001
“Other”***	189 (23%)	180 (25%)	9 (8%)	<0.001
Missing data	0	0	0	
SAP (mmHg)	135 ± 36	131 ± 33	154 ± 47	<0.001
SAP				
Hypertension >140 mmHg	334 (41%)	263 (37%)	71 (66%)	<0.001
Normotension 90 to 140 mmHg	411 (50%)	386 (54%)	25 (23%)	<0.001
Hypotension <90 mmHg	74 (9%)	62 (9%)	12 (11%)	<0.001
Missing data	3	1	2	
GCS	15 [15]	15 [15]	9 [4-15]	<0.001
GCS				
>13	684 (84%)	648 (92%)	36 (33%)	<0.001
9 to 13	65 (8%)	45 (6%)	20 (19%)	<0.001
≤8	64 (8%)	12 (2%)	52 (48%)	<0.001
Missing data	9	7	2	
Admission INR	4.7 ± 3.4	4.7 ± 3.5	4.4 ± 2.7	0.236
Normal (≤1.5)	45 (5%)	40 (6%)	5 (5%)	0.385
Therapeutic (>1.5 to 4)	394 (48%)	341 (48%)	53 (48%)	
Supratherapeutic (>4)	345 (42%)	300 (42%)	45 (41%)	
Missing value	38 (5%)	31 (4%)	7 (6%)	
VKA reversal treatment				
No guideline-concordant				
Level 0	361 (44%)	299 (42%)	62 (56%)	0.06
Level 1	103 (13%)	92 (13%)	11 (10%)	
Level 2	34 (4%)	32 (5%)	2 (2%)	
Guideline-concordant				
Level 3	90 (11%)	85 (12%)	5 (5%)	
Level 4	217 (26%)	190 (27%)	27 (25%)	
VKA reversal treatment				
Not guideline-concordant (Levels 0 + 1 + 2)	509 (62%)	432 (61%)	77 (70%)	0.06
Guideline-concordant (Levels 3 + 4)	313 (38%)	280 (39%)	33 (30%)	

Data are mean ± SD, median [25^th^ to 75^th^ percentiles], or number (percentage). *P-*values refer to comparison between alive and dead patients at Day 7.

INR*,* international normalized ratio; SAP*,* systolic arterial blood pressure; GCS*,* Glasgow coma scale; VKA*,* vitamin K antagonist.

*The sum is higher than 100% because two indications can be established for the same patient.

**Patients with no antiplatelet treatment or no history of severe hemorrhage are deducted from the presented results.

***“Other” types include hemopericardium (*N* = 1), hemothorax (*N* = 15), vascular bound (*N* = 4), emergency procedures (*N* = 126) and shock or red blood cells transfusion for epistaxis (*N* = 55), hematuria (*N* = 34) or wound of the scalp (*N* = 4).

VKA reversal treatment comprised PCC in 509 patients (62%) but only 379 (46%) received the recommended doses (≥20 IU/kg). Only 27 patients (3%), including 2 in the ICH subgroup, received FFP. Vitamin K was administered in 583 patients (71%) and the recommended dose (≥5 mg) was used in 531 patients (65%). The combination of PCC (≥20 IU/kg) and vitamin K (≥5 mg), regardless of administration time, was given to 336 patients (41%). Guideline-concordant administration of PCC was uncertain in 15 patients (total dose of PCC without the patient’s body weight), and two experts agreed on 13 cases (80% agreement with a κ of 0.60). Finally, treatment was considered concordant with guidelines in 313 patients (38%).

**Figure 2:**
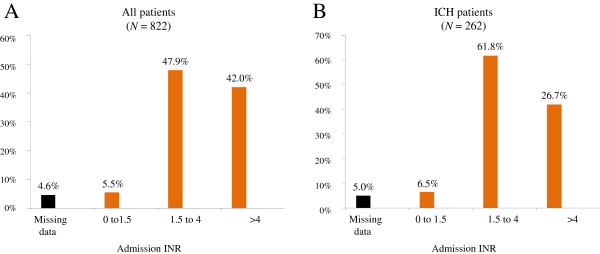
**Admission INRs in the whole cohort (A) and in the ICH subgroup (B).** Most patients were in the therapeutic range (INR between 1.5 and 4). ICH, intracranial hemorrhage; INR, international normalized ratio.

Admission INRs are shown in Figure 2. Post-reversal INR values were available for 417 patients (51%); however, they were obtained within one hour in only 61 (7%) patients and within six hours in 225 (27%) patients. Considering all post-reversal INR that we had collected, the proportion of patients in which coagulation was normalized (post-reversal INR ≤1.5) was significantly greater with GC-PCC-K. Nobody received a complementary PCC dose in case of post-reversal INR >1.5.

In multivariate analysis, we observed that early mortality was significantly associated with no GC-PCC-K in the whole cohort (OR = 2.15 (1.20-3.88); *P* = 0.011) and ICH patients (OR = 3.23 (1.53-6.79); *P* = 0.002) (Table 2). Using bootstrapping, the OR of no guideline-concordant treatment was 2.44 (1.09-5.74) in the whole cohort leading to an optimism of 0.24.

**Table 2:** Multivariable analysis of early (seven-day) mortality

**Variable**	**All patients (*N*** **= 822)**			**ICH (*N*** **= 262)**		
	***N***	**OR (95% CI)**	***P***	***N***	**OR (95% CI)**	***P***
Type of hemorrhage	822					
“Other”*	189	1.0				
Gastrointestinal	264	0.61 (0.22 to 1.70)	NS			
Deep-muscle	107	0.51 (0.14 to 1.93)	NS			
ICH	262	5.05 (1.97 to 12.94)	<0.001			
Age (years)	822			262		
≤65 years	129	1.0		34	1.0	
66 to 75	144	1.01 (0.35 to 2.93)	NS	47	2.32 (0.57 to 9.47)	NS
76 to 85	368	1.67 (0.68 to 4.10)	NS	122	2.71 (0.78 to 9.34)	NS
>85	181	2.21 (0.84 to 5.77)	NS	59	3.79 (1.01 to 14.26)	0.049
Admission INR	822			262		
≤1.5	45	1.0		17	1.0	
No INR	38	0.76 (0.12 to 4.71)	NS	13	1.01 (0.11 to 9.71)	NS
>1.5 to 4	394	1.12 (0.33 to 3.80)	NS	162	1.40 (0.32 to 6.19)	NS
>4	345	1.36 (0.38 to 4.87)	NS	70	1.56 (0.31 to 7.81)	NS
GCS score	813			261		
>13	684	1.0		149	1.0	
9 to 13	65	3.67 (1.80 to 7.51)	<0.001	51	4.23 (1.87 to 9.53)	<.001
≤8	64	30.35 (13.43 to 68.61)	<0.001	61	39.81 (15.70 to 100.91)	<.001
SAP	819			262		
90 to 140 mmHg	411	1.0		64	1.0	
<90 mmHg	74	7.91 (3.06 to 20.44)	<0.001	3	0.67 (0.01 to 33.01)	NS
>140 mmHg	334	1.15 (0.58 to 2.28)	NS	195	1.29 (0.55 to 3.06)	NS
VKA reversal	822			262		
Guideline concordant	313	1.0	0.011	116	1.0	.002
Not guideline-concordant	509	2.15 (1.20 to 3.88)		146	3.23 (1.53 to 6.79)	
		Hosmer–Lemeshow Goodness-of-Fit Test: Pr >χ^2^ = 0.99			Hosmer–Lemeshow Goodness-of-Fit Test: Pr >χ^2^ = 0.52	
		c-statistic = 0.89			c-statistic = 0.86	

CI, confidence interval; ICH*,* intracranial hemorrhage; GCS*,* Glasgow coma scale; NS, not statistically significant; OR, odds ratio; SAP, systolic arterial blood pressure; trt: treatment.

*“Other” types include hemopericardium (*N* = 1), hemothorax (*N* = 15), vascular bound (*N* = 4), emergency procedures (*N* = 126) and shock or red blood cell transfusion for epistaxis (*N* = 55), hematuria (*N* = 34) or a wound of the scalp (*N* = 4).

Within the first week after admission, 10 thromboembolic events with five associated deaths were recorded: pulmonary embolism (*N* = 5), cerebral or coronary ischemic events (*N* = 3), venous (*N* = 1) and peripheral artery thrombosis (*N* = 1). Hemorrhagic recurrence was observed in 83 patients (11%) with four associated deaths, including 14 patients with two deaths in the ICH subgroup.

## Discussion

This is the first study showing that GC-PCC-K (that is, administration of recommended doses of PCC and vitamin K) within eight hours after patient admission for severe hemorrhage is associated with a decrease in seven-day mortality. We found a two-fold decrease in mortality (OR = 2.2 (1.2-3.9); *P* = 0.011) when guideline-concordant treatment was performed. Mortality among ICH patients has been reported to be approximately 20 to 30% in non-anticoagulated patients and 40 to 65% in VKA-treated patients [5,17]. In this study, GC-PCC-K was associated with a three-fold reduction of mortality in the ICH subgroup (OR = 3.2 (1.5 to 6.8); *P* = 0.002), suggesting that recommended doses of PCC and vitamin K administered in the first eight hours, can decrease mortality to levels close to those observed in non-anticoagulated ICH patients. The very low mortality rate (4%) observed in patients with other types of severe hemorrhage does not allow us to draw conclusions with regard to these patients due to lack of statistical power. Hypotension was associated with increased mortality in the whole cohort, but this association was not observed in ICH patients. GCS was also strongly associated with early mortality. As a result of population ageing, the prevalence of VKA-related hemorrhagic complications (ICH in particular) increases worldwide [1,5]. The median age of 80 years in our study is a clear reminder of this fact. However, we did not observe a statistically significant association between age and mortality.

Guideline-concordant treatment doses for normalization of coagulation and a fixed time frame within which reversal is considered useful are probably the two conditions required to improve prognosis. To achieve a post reversion INR ≤1.5, most pharmaceutical companies recommend a PCC dose related to admission INR value. However, INR is not a linear representation of the coagulation and a dose-response curve has not been demonstrated in the literature [18,19]. Moreover, in the absence of bedside INR monitoring, waiting for an admission INR value from the laboratory in bleeding patients may result in increased blood losses and coagulation factor consumption. Based on a previous small cohort study of VKA-treated patients with ICH suggesting that a single-regimen dose of PCC (administered as an intravenous bolus) is effective in achieving a post-reversal INR ≤1.5, French guidelines recommend, in order to save time when admission INR is not available, the administration of a single-regimen dose of 25 IU/kg factor IX equivalent PCC (that is, 1 mL/kg with available four-factor PCCs) in combination with 10 mg of vitamin K [9,10]. Use of the recommended single-regimen dose has not yet become routine practice, as shown by the fact that only 205 patients (25%) received a dose of 25 IU/kg in our study. Guidelines also strongly recommend measuring the INR after reversal to control the degree of reversal and further normalize the coagulation 30 minutes after the first administration if the PCC dose was insufficient [10]. Unfortunately, post-reversal INR was obtained within less than one hour in only 7% of our patients, underscoring the need for improved guideline adherence. In our study, bedside INR monitoring was never used. Adoption of this technique might help increase the rate of post-reversal INR measurement within one hour after treatment administration.

Time between hemorrhage onset and VKA reversal is a key variable to be considered when evaluating treatment efficacy. In patients with ICH, hematoma volume is crucial for the prognosis and is known to increase during the first day following admission [20]. Not surprisingly, it has been shown that late reversal, over eight hours after admission, does not modify the outcome [5]. Based on a previous study, we chose a time frame of eight hours to define timely treatment, which was considered realistic taking into consideration the time needed to confirm the diagnosis of hemorrhage, which is not always easy, for example, in patients with deep-muscle hematoma, gastrointestinal hemorrhage or ICH.

Fear of thrombosis may be one of the reasons for not performing VKA reversal, particularly in patients (6%) with a normal initial INR (≤1.5) and a potentially high thrombotic risk if the single-regimen approach is considered. In severely bleeding patients with a high thromboembolic risk, total mortality (*N* = 110, 13%) was mainly related to hemorrhage, with only five deaths (0.6%) observed in patients with a thromboembolic event [21]. This provides a good example of the risk balance and reinforces the fact that the priority is to organize a rapid reversal as soon as the diagnosis of severe hemorrhage is confirmed.

Some limitations of our study deserve consideration. First, we studied mortality within seven days as it appears to be the period when the cause of death is a direct consequence of hemorrhage in all situations, even in ICH patients [22]. In a study of ICH-related mortality in warfarin users and non-users, Huhtakangas *et al*. observed a two-fold difference between death rates during the first week after stroke onset and no further increase after one week with parallel mortality curves [3]. We considered that many clinical complications or medical attitudes not related to VKA reversal could interfere with 30-day mortality in all groups. Second, we did not exclude patients with care limitation decisions, although such decisions are not rare in an aged population with severe neurological insults, such as in ICH, and may also be associated with “self-fulfilling prophecies” [23]. In an observational study conducted in emergency conditions it was not possible to clearly identify the timing of these decisions in relationship with VKA reversal and to precisely distinguish explicit and implicit decisions during this early phase.

It is interesting to consider that only 41% of patients received the recommended doses of PCC and vitamin K and only 38% of them did so within the first eight hours after the onset of bleeding. These results were similar to those of a previous study [16]. A large French study, which was performed in 33 hospitals during 2008 to 2011 showed the same difficulties in obtaining good guideline adherence for VKA reversal in patients with severe hemorrhage [24]. More important, in our study, only 27% of patients who had received PCC had an INR post-reversion during the six hours. It appears therefore necessary to promote guideline dissemination and implementation. It would also be beneficial to improve the current guidelines by including explicit time frames for treatment administration.

## Conclusion

Guideline-recommended doses of PCC and vitamin K were associated with a reduced mortality in patients on VKA therapy presenting with severe hemorrhage, particularly among patients with ICH in whom we observed a three-fold decrease in mortality. VKA reversal should be initiated within eight hours after admission. Only 38% of our patients received appropriate treatment, indicating the need for improved guideline adherence.

## Key messages

● In patients on VKA therapy presenting with severe hemorrhage, international guidelines recommend, as soon as the diagnosis is confirmed, the administration of PCC (≥20 UI/kg) and vitamin K (≥5 mg) to normalize coagulation (post-reversal INR ≤1.5).

● A guideline-concordant administration dose of PCC and vitamin K administrated in the first eight hours was associated with a two-fold decrease in seven-day mortality overall and with a three-fold decrease in the ICH subgroup

● The guideline-concordant reversal was performed in 38% of the patients within eight hours after admission

● Whereas pre-reversal INR is not absolutely necessary, post-reversal INR is essential to evaluate treatment efficacy

● The post-reversal INR target must be performed systematically and immediately after PCC administration

## Abbreviations

CI: Confidence interval; EPAHK: Evaluation pronostique de l’antagonisation des hémorragies graves sous AVK; FFP: Fresh frozen plasma; GC-PCC-K: Guideline-concordant of administration PCC and Vitamin K; GCS: Glasgow coma scale score; HR: Hazard ratio; ICH: intracranial hemorrhage; INR: International normalized ratio; OR: Odds ratio; PCC: Prothombin concentrated complex; SAP: Systolic arterial blood pressure; VKA: Vitamin K antagonist.

## Competing interests

KT has received consulting and speaker’s fees from Sanofi, Lilly and LFB. BR has received travel grants, consultancy fees, honoraria and study grants from Sanofi, GlaxoSmithKline, ThermoFisher, Octapharma, Sangart and LFB. BT report has received consulting fees from LFB. C.-MS has received grant monies (industry-related sources) from NovoNordisk, CSL Behring and LFB, and speaker’s fees from CSL Behring and LFB. EV has received travel grants, consultancy fees, honoraria and study grants from Pfizer, Eli Lilly, Sanofi, Abbott, Fresenius, Stallergene, Boehringer Ingelheim, Merk and LFB. BV has received grant monies (industry-related sources) from ThermoFisher and speaker’s fees from ThermoFisher, CSL Behring, Octapharma and LFB.

## Author contributions

KT and BV contributed to the conception and design of the study, data collection and analysis, manuscript writing and final approval of the manuscript. BR contributed to the conception and design of the study, manuscript writing and final approval of the manuscript. BT, EV and CMS contributed to the conception and design of the study, revising the draft for important intellectual content, and final approval of the manuscript. All authors read and approved the final manuscript.

## Scientific committee

Prof Bruno Riou, MD, PhD; Prof Charles-Marc Samama, MD, PhD, FCCP; Dr Karim Tazarourte, MD, PhD; Dr Benjamin Tremey, MD; Prof Eric Vicaut, MD, PhD; Dr Bernard Vigué, MD, PhD; Philippe Richardot (Advanced Drug Development Service, Montpellier, France) performed the statistical analysis.

Local investigators (study group) and participating centers (emergency departments) are listed according to the number of patients included in the study (by decreasing order):

Prof Luc-Marie Joly, CHU Charles Nicolle, 76000 Rouen (83 patients)

Prof Thibault Desmettre, CHU Jean Minjoz, 25030 Besançon (69 patients)

Dr Louis Soulat, CH, 36019 Chateauroux (65 patients)

Prof Jeannot Schmidt, CHU Gabriel Montpied, 63003 Clermont-Ferrand (64 patients)

Prof Frédéric Adnet, CHU Avicenne, AP–HP, 93009 Bobigny (45 patients)

Dr Pierre Taboulet, CHU Saint Louis, AP–HP, 75010 Paris (38 patients)

Dr Christine Beril-Vallejo, CHU Dupuytren, 87402 Limoges (36 patients)

Dr David Emmanuelle, CH Melun, 77000 Melun (35 patients)

Dr Mustapha Sebbane, CHU Hôpital Lapeyronie, 34090 Montpellier (33 patients)

Dr François Braun, Hôpital Bon Secours, 57038 Metz (27 patients)

Dr Lionel Bertrand, CHG de Montauban, 82013 Montauban (25 patients)

Dr Jacques Choukroun, CH du Mans, 72037 Le Mans, (23 patients)

Prof Bertrand Renaud; CHU Henri Mondor, AP–HP, 94000 Créteil (20 patients)

Dr Samuel Delerme, CHU Pitié-Salpêtrière, AP–HP, 75013 Paris (20 patients)

Prof Jacques Bouget, CHU Pontchaillou, 35033 Rennes, (20 patients)

Prof Pierre-Marie Roy, CHU d’Angers, 49933 Angers (17 patients)

Dr Armelle Lucas Amichi, Hôpital Antoine Béclère, AP–HP, 92141 Clamart (17 patients)

Prof Françoise Carpentier, CHU Grenoble – Hôpital Nord, 36000 Grenoble (15 patients)

Dr Didier Honnart, CHU – Hôpital général, 21033 Dijon (14 patients)

Prof Christine Ammirati, CHU Amiens – Hôpital Nord, 80054 Amiens (14 patients)

Dr Catherine Legall, CH Victor Dupouy, 95107 Argenteuil (14 patients)

Prof Jean-Stéphane David, CHU Lyon Sud, 69007 Pierre-Benite (13 patients)

Dr François Lecomte, CHU Cochin, AP–HP, 75014 Paris (13 patients)

Dr Dominique El Kouri, CHU – Hôtel Dieu, 44093 Nantes (11 patients)

Dr Jacqueline Depret-Vassal, CHU Bicêtre, AP–HP, 94275 Le Kremlin Bicêtre (10 patients)

Prof Eric Wiel, CHRU de Lille, 59000 Lille (10 patients)

Prof Jean-Emmanuel de La Coussaye, CHU Carrémeau, 30029 Nimes (10 patients)

Dr Sophie Fernandez, CHU Purpan, 31059 Toulouse (9 patients)

Dr Jacques Remize, CH de Brive, 19100 Brive, (8 patients)

Dr Etienne Hinglais, Hôpital Tenon, AP–HP, 75020 Paris (6 patients)

Dr Annick Genty, CHU Edouard Herriot, 69437 Lyon (6 patients)

Dr Daniel Epain, CHG Lagny, 77405 Lagny-sur-Marne (5 patients)

Dr Maurice Raphaël, CHG, 93370 Montfermeil (5 patients)

Dr Bruno Goulesque, Hôpital Emile Muller, 68100 Mulhouse (5 patients)

Dr Christelle Dejou, CH, 15002 Aurillac (5 patients)

Dr Leila Lavagna-Perez, CHU Beaujon, AP–HP, 92110 Clichy (4 patients)

Dr Erick Duret, Hôpital Saint Nicolas, 55107 Verdun (4 patients)

Dr Mahmut Gundesli, Hôpital Central, 54035 Nancy (4 patients)

Dr Hacène Moussouni, CH de Tourcoing, 59200 Tourcoing (3 patients)

Prof Dominique Pateron, CHU Saint Antoine, AP–HP, 75012 Paris (2 patients)

Dr Patrick Miroux, CHG, 60200 Compiègne (2 patients)

Dr Patrick Deschamps, CHG Pontoise 95300 Pontoise (2 patients)

Dr Cécile Chassaignon, CHG, 93143 Bondy (1 patient)

Dr Arnaud Delahaye, CHG, 12000 Rodez (1 patient)

The funder (LFB Biomédicament, Les Ulis, France) had a role in the conduct of the study and data collection but was not involved in study design, analysis of the data, writing of the report and the decision to submit for publication. The authors had access to all study data and made the final decision to submit the manuscript for publication.
